# Identification of new HLA-A*0201-restricted cytotoxic T lymphocyte epitopes from LDHC in lung adenocarcinoma

**DOI:** 10.3389/fimmu.2025.1564731

**Published:** 2025-04-09

**Authors:** Ruifang Zhong, Xiaohong Guo, Chuncai Wu, Yangyi Guo, Yanli Kang, Jianbin You, Falin Chen, Qianshun Chen, Liangyuan Chen

**Affiliations:** ^1^ Shengli Clinical Medical College of Fujian Medical University, Fuzhou, China; ^2^ Department of Clinical Laboratory, Zhangzhou Affiliated Hospital of Fujian Medical University, Zhangzhou, China; ^3^ Department of Clinical Laboratory, The Third Hospital Of LongYan, LongYan, China; ^4^ Department of Clinical Laboratory, Fuzhou University Affiliated Provincial Hospital, Fuzhou, Fujian, China; ^5^ Department of Thoracic Surgery, Fuzhou University Affiliated Provincial Hospital, Fuzhou, Fujian, China

**Keywords:** lung adenocarcinoma, HLA-A2, lactate dehydrogenase C, recombinant protein, peptides

## Abstract

**Background:**

Lactate dehydrogenase C (LDHC) is a kind of cancer-testis antigen (CTA) that has been reported to be a biomarker for diagnosis, efficacy evaluation, and recurrence monitoring of lung adenocarcinoma (LUAD). This study aims to assess the value of LDHC in peptide-based vaccines for LUAD immunotherapy.

**Methods:**

The LDHC recombinant protein was purified and its effect on PC9 cells was evaluated by wound healing assay, Transwell invasion, and migration assay. Ten HLA-A2-restricted LDHC-derived peptides were predicted and synthesized, and the affinity for the HLA-A2 molecule was analyzed by T2 binding assay and molecule docking. Enzyme-linked immunospot (ELISpot) and LDH cytotoxicity assay were performed to determine the interferon-γ (IFN-γ) release level and tumor cell lysis ability of peptide-induced specific cytotoxic T lymphocytes (CTLs).

**Results:**

The LDHC recombinant protein promoted invasion and migration of PC9 cells. Three HLA-A2-restricted LDHC-derived peptides P2 (LDHC^170–180^, FRYLIGEKLGV), P5 (LDHC^116–124^, IMKSIIPAI), and P6 (LDHC^172–180^, YLIGEKLGV) had high affinity for the HLA-A2 molecule at 50 μg/mL. P6 (LDHC^172–180^, YLIGEKLGV) elicited the strongest IFN-γ-secreting cytotoxic T lymphocyte (CTL) response and exhibited potent cytotoxicity against HLA-A2-positive cells with high LDHC expression.

**Conclusions:**

LDHC may serve as a targetable biomarker for peptide-based immunotherapy of LUAD.

## 1 Introduction

Lung cancer remains the leading cause of cancer-related death worldwide, with an estimated 2.4 million new cases and 1.8 million deaths reported in 2022. Non-small cell lung cancer (NSCLC) constitutes approximately 85%, or roughly 2.109 million cases. The mortality rate of NSCLC represents 18.7% of all global lung cancer deaths, resulting in approximately 0.34 million fatalities annually. Among the NSCLC subtypes, lung adenocarcinoma (LUAD) is the most prevalent (40%–50%), followed by squamous cell carcinoma (20%–30%) and large cell carcinoma (10%–15%) (1, 2). Statistical data showed that lung cancer has the highest mortality rate and the lowest 5-year survival rate among cancers (25%) (3). Therefore, effective therapy for lung cancer has always been a hot topic. Molecular targeted therapy plays a crucial role in the treatment strategy for NSCLC (4). However, drug resistance limits its effectiveness. Therefore, there is an urgent need to identify more effective molecular targets.

With high immunogenicity and restricted expression, cancer/testis antigens (CTAs) are considered good candidate targets for tumor vaccines and T-cell-targeted therapies (5). Lactate dehydrogenase C (LDHC) is one of the CTAs belonging to the lactate dehydrogenase (LDH) family (6). LDHC is the first lactate dehydrogenase isoform restricted to tumor cells and prefers lactate as a substrate compared with other LDH isoenzymes (7). To date, LDHC has been found to be highly expressed in tumors such as lung cancer, melanoma, breast cancer, hepatocellular carcinoma, and prostate cancer (8–11). Furthermore, Naik and Decock reported that knockdown of LDHC expression increased DNA damage and apoptosis in breast cancer cells (12). Zhaolei Cui et al. performed survival analysis and Cox regression analysis. Their study showed that in hepatocellular carcinoma patients, high expression of LDH-C4 was correlated with poor prognosis, and LDH-C4 level was an independent risk factor (11). Furthermore, compared to the initial diagnosis, LDHC mRNA levels in serum and exosomes increased post-treatment but decreased in the recurrence group (11). However, LDHC is a protein-coding gene, and the role of the LDHC protein is still unknown.

Due to the limitations of traditional chemotherapeutic drugs, which include low target specificity and low bioavailability, more effective antitumor therapies urgently need to be developed. Consequently, peptide-based therapeutic tumor vaccines may become an important method in tumor immunotherapy for its advantages, such as convenience, low cost, and low carcinogenic potential. LDHC has been applied to peptide-based therapeutic research. Fausto Petrelli et al. identified two HLA−A*0201 immunogenic epitopes of LDHC (LDHC^41−55^ and LDHC^288−303^), which increased interferon-γ (IFN-γ) secretion by CD8^+^ T cells and cancer cell killing of HLA-A*0201/LDHC-positive breast cancer cells (13). However, the evidence for LDHC in peptide-based treatment of cancers is still insufficient. Our previous study showed that the expression of LDHC in LUAD tissues was significantly upregulated compared to normal tissues and promoted tumor progression in LUAD cells through the PI3K/Akt/GSK-3β pathway (14). In this study, we aim to explore the impact of LDHC recombinant protein on the biological behavior of LUAD and the immunogenicity of HLA-A2-restricted LDHC peptides.

## 2 Methods

### 2.1 Cell lines, peripheral blood mononuclear cells, and HLA-A2 expression

Three LUAD cell lines (A549, H1792, PC9) and T2A2 cell lines were stored in the laboratory’s liquid nitrogen tank. All cells were cultured in complete RPMI 1640 medium (BasalMedia, Shanghai, China) supplemented with 10% fetal bovine serum (FBS, Thermo Fisher Scientific, United States) and 1% penicillin–streptomycin solution (P/S, BasalMedia, Shanghai, China) at 37°C and 5% CO_2_.

Peripheral blood mononuclear cells (PBMCs) were isolated from a healthy, HLA-A2-positive volunteer and cultured in complete RPMI 1640 medium supplemented with 10% FBS and 1% P/S at 37°C and 5% CO_2_. The expression of HLA-A2 was confirmed by flow cytometry with an HLA-A2 Monoclonal Antibody (BB7.2), PE-Cyanine7 (Thermo Fisher Scientific, United States), to select HLA-A2-positive blood donors and target cancer cell lines.

### 2.2 Prediction and synthesis of LDHC-derived peptides and LDHC recombinant protein

Peptides derived from LDHC were predicted by the SYFPEITHI (http://www.syfpeithi.de/) and NetMHC 4.0 (https://services.healthtech.dtu.dk/services/NetMHC-4.0/) servers. The CMV peptide NLVPMVATV served as a positive control. Ten peptides with a high score on an HLA-A2 molecule were synthesized. The purity of the peptides (>95%) was determined by analytical high-performance liquid chromatography (HPLC) and mass spectrometry (MS) analysis.

The protein structure and function prediction of the LDHC recombinant protein was performed by the I-TASSER server (https://zhanggroup.org/I-TASSER/). The predicted structure of the LDHC recombinant protein was further refined using the GalaxyRefine server (https://galaxy.seoklab.org/cgi-bin/submit.cgi?type=REFINE) to improve its stereochemical quality.

### 2.3 RT-qPCR analysis

Total RNAs were extracted from cells using the TRIzol^®^ reagent (Thermo Fisher Scientific, United States), according to the manufacturer’s instruction. Briely, homogenize cells in TRIzol® (5–10 × 10^6^ cells) and incubate for 5 min at room temperature. Add chloroform (0.2 mL per 1 mL TRIzol®), shake vigorously, and centrifuge at 12,000 × g for 15 min at 4°C. Transfer the aqueous phase to a new tube, mix with isopropanol (0.5 mL per 1 mL TRIzol®), and centrifuge at 12,000 × g for 10 min at 4°C to pellet RNA. Wash the pellet with 75% ethanol, centrifuge at 7,500 × g for 5 min, air-dry briefly, and dissolve in RNase-free water. Total RNA was reverse transcribed into cDNA using the TaKaRa PrimeScript™ RT reagent kit (TaKaRa Biotechnology, Japan) following the manufacturer’s instructions. Briefly, RNA template, oligo dT primer, random hexamers, and dNTPs were mixed with PrimeScript RT enzyme and buffer, incubated at 37°C for 15 min, then 85°C for 5 sec to inactivate the enzyme. The resulting cDNA was stored at -20°C for subsequent PCR analysis. Cell RNAs were reverse transcribed into cDNAs using the TaKaRa PrimeScrip™ RT reagent kit (TaKaRa Biotechnology, Japan) according to the manufacturer’s instructions. The primer sequences of LDHC-F and LDHC-R were 5′-TCATTCCTGCCATAGTCCA-3′ and 5′-CAATTACACGAGTTACAGGTA-3′. The primer sequences of GAPDH were 5′-TGACTTCAACAGCGACACCCA-3′ and 5′-CACCCTGTTGCTGTAGCCAAA-3′. LDHC mRNA levels were determined using NanoDrop 2000. Reaction was performed using a fluorescence quantitative PCR detector and TB Green^®^Premix Ex Taq™ Green II (TaKaRa Biotechnology, Japan) at the following conditions: 40 cycles of denaturation at 95°C for 10 min, followed by 95°C for 15 s, with extension at 60°C for 1 min. Subsequently, the relative expressions of LDHC mRNAs were calculated using the 2^−ΔΔCt^ method normalized to GAPDH.

### 2.4 Wound healing assay

Cells (1 × 10^6^) were seeded in six-well plates and cultured until they reached 100% confluence. Consequently, a 200-μL pipette tip was used to draw a straight scratch across the cell monolayer. The medium was replaced with fresh RPMI 1640 containing LDHC recombinant protein at concentrations of 0 ng/mL, 100 ng/mL, and 200 ng/mL. The wells were gently washed with PBS to remove cell debris. Subsequently, the plate was placed inside an incubator at 37°C and 5% CO_2_. Photos were taken and observed at 24 or 48 h.

### 2.5 Migration assay

A total of 200uL of RPMI 1640 medium containing 5 × 10^4^ cells were seeded into the top chambers of the Transwell plates with serum-free RPMI 1640 medium, and 600 μL containing LDHC recombinant protein (0 ng/mL, 100 ng/mL, and 200 ng/mL) was added into the lower chambers. Cells were incubated for 36 h at 37°C and 5% CO_2_. Finally, the cells were fixed with 75% ethanol and stained with crystal violet.

### 2.6 Invasion assay

Matrigel (Corning, Japan) was thawed overnight at 4°C, then kept on ice. Using a precooled pipette tip, 50 µL of Matrigel was evenly spread onto the polycarbonate membrane of the Transwell upper chamber. The coated chamber was incubated at 37°C for 30 min to allow gel polymerization. Subsequent steps followed the standard migration assay protocol.

### 2.7 HLA-A2 molecule docking

The X-ray crystal structures of 3V5K (PDB: 3V5k) were obtained from the Protein Data Bank (https://www.rcsb.org/). The compounds were expanded to 3D structures using Open Babel at pH = 7.4 protonation state (15). The receptor protein and ligands were prepared and parameterized using AutoDock Tools (ADT3). Docking grid files were created with AutoGrid, and molecular docking simulations were performed using AutoDock Vina (version 1.2.0) (16, 17). The most favorable docking pose was chosen for interaction analysis, and the resulting protein–ligand interactions were visualized using PyMOL. The 2D plots of the protein–ligand interactions was analyzed using the LigPlot software (version 2.2) (18).

### 2.8 T2A2 binding assay

T2 cells were used to evaluate the binding and stabilization of HLA-A2 molecules stimulated by LDHC-derived peptides. T2 cells were stripped in PBS for 2 h, washed, and resuspended in a serum-free culture medium. A total of 2 ×10^5^ cells were incubated with 3 mg/mL of β2-microglobulin (β2-M, Merck, United States) and peptide (2 μg/mL, 10 μg/mL, 50 μg/mL, 100 μg/mL, or 250 μg/mL) in a final volume of 200 μL for 4 h at 37°C. Cells were then washed and stained with the PE-conjugated HLA-A2 monoclonal antibody (clone: BB7.2, Dakewe Biotech, Beijing) before cytometry evaluation (FACSCalibur flow cytometer Becton Dickinson, United States). The fluorescence intensity (FI) was calculated as follows: FI = (mean fluorescence intensity (MFI) of peptide-pulsed T2 cells/MFI of non-peptide-loaded T2 cells) − 1. Peptides with an FI >1.5 were regarded as high-affinity candidates.

### 2.9 Enzyme-linked immunospot assay

PBMCs were isolated from the peripheral blood of healthy donors, and cytotoxic T lymphocytes (CTLs) were expanded according to our previous protocol (18). A human IFN-γ precoated enzyme-linked immunospot (ELISpot) kit (DAKEWE, China) was used for the ELISpot assay. Briefly, 5 × 10^5^ cells induced from dendritic cells (DCs) indicated that peptides were added to a 96-well PVDF membrane plate coated with anti-IFN-γ antibody. Spots were developed using a 3-amine-9-ethylcarbazole (AEC, sigma-Aldrich, United States) solution and counted using an ELISpot reader (Mabtech IRIS™ FluoroSpot/ELISpot reader, Sweden).

### 2.10 CTL killing assay

The cytotoxicity of peptide-specific T cells stimulated by peptides was tested using the LDH Cytotoxicity Assay Kit (Beyotime Biotechnology, China). The EasySep™ Human CD8 Positive Selection Kit II (STEMCELL Technologies, Canada) was used to isolate CD8^+^ cells from PBMCs. A no-cell control (only culture medium), an untreated control (cells treated with the same amount of solvent), and a control for maximum LDH release were set. Target cells (5 × 10³ per well) and effector cells were added at effector-to-target (E:T) ratios of 10:1, 20:1, and 40:1. The plates were incubated at 37°C, 5% CO_2_ for 4–6 h, followed by centrifugation at 200×*g* for 10 min. One hundred fifty microliters of the supernatant was aspirated from each well and transferred to a new 96-well plate. Ten microliters of 0.4 mol/L lactic acid solution, 20 μL of 4 mmol/L 2-p-iodophenyl-3-p-nitrophenyl tetrazolium, and 20 μL of reaction solution [containing 0.03% BSA, 2.7 U/ml of diaphorase, 4.5 mmol/L of reduced coenzyme I (NAD^+^), and 1.2% sucrose in PBS] were added to each well and incubated at room temperature for 20 min. The optical density (OD value) of each well was measured with a detection wavelength of 490 nm and a reference wavelength of 650 nm. The percentage of specific lysis was calculated as follows: Specific lysis (%) = (OD of the experimental group − OD of total spontaneous release)/(OD of the maximum release group − OD of total spontaneous release) × 100%.

### 2.11 Statistical analysis

Data were analyzed with GraphPad Prism 9.5.0 and are shown as the mean ± standard deviation (SD). Student’s *t*-test was used for statistical comparisons, with a *p*-value <0.05 considered statistically significant.

## 3 Results

### 3.1 LDHC recombinant protein promotes LUAD cell growth

**Figure 1:**
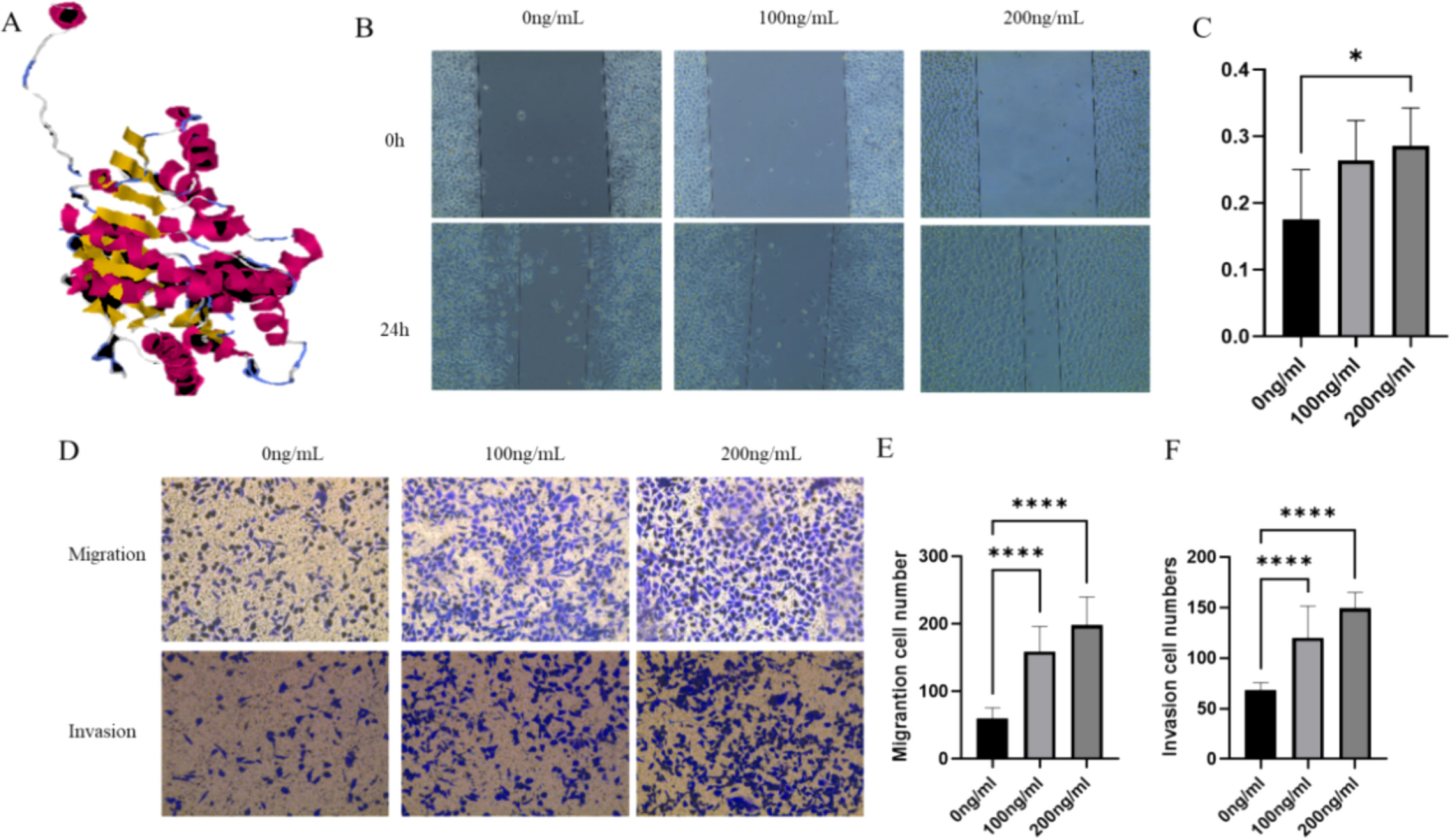
LDHC recombinant protein promotes LUAD cell growth. **(A)** The 3D model of the LDHC recombinant protein. **(B, C)** The wound healing assay indicated that the migration rate of the 200-ng/mL group was higher than that of the 0-ng/mL group. **(D–F)** Transwell assays showed that the migration and invasion of cell numbers in 100 ng/mL and 200 ng/mL of LDHC recombinant protein were higher than those of the 0-ng/mL group (*p < 0.05; ****p < 0.0001).

The expression region of the LDHC recombinant protein was found to be 58–479 amino acids, with some active site residues included. The 3D model was generated by the I-TASSER server with a C-score of 1.51, an estimated TM score of 0.92 ± 0.06, and an estimated RMSD of 3.3 ± 2.3 Å, indicating that the predicted protein structure was processed with high reliability and accuracy (**Figure 1A**). The GalaxyRefine server generated five protein models, among which MODEL 2 was found to be the optimal choice due to its lowest MolProbity score (2.013), highest Rama favored value (95.4%), relatively low clash score (15.6), and high GDT-HA value (0.9721), indicating superior stereochemical quality, favorable dihedral angles, fewer atomic clashes, and a strong global similarity to the original structure (**Supplementary Table 1**).

The wound healing assay indicated that the migration rate of the 200-ng/mL group was higher than that of the 0-ng/mL group (*p* = 0.033) (**Figures 1B, C**). The Transwell assays were used to assess the migration and invasion ability when LUAD cells were co-cultured with different levels of LDHC recombinant protein. **Figures 1D–F** show that the migration and invasion cell numbers in 100 ng/mL and 200 ng/mL of LDHC recombinant protein were higher than those of the 0-ng/mL group (all with *p* < 0.0001).

### 3.2 Screening of high-affinity peptides using the T2 affinity test and concentration gradient test

**Figure 2:**
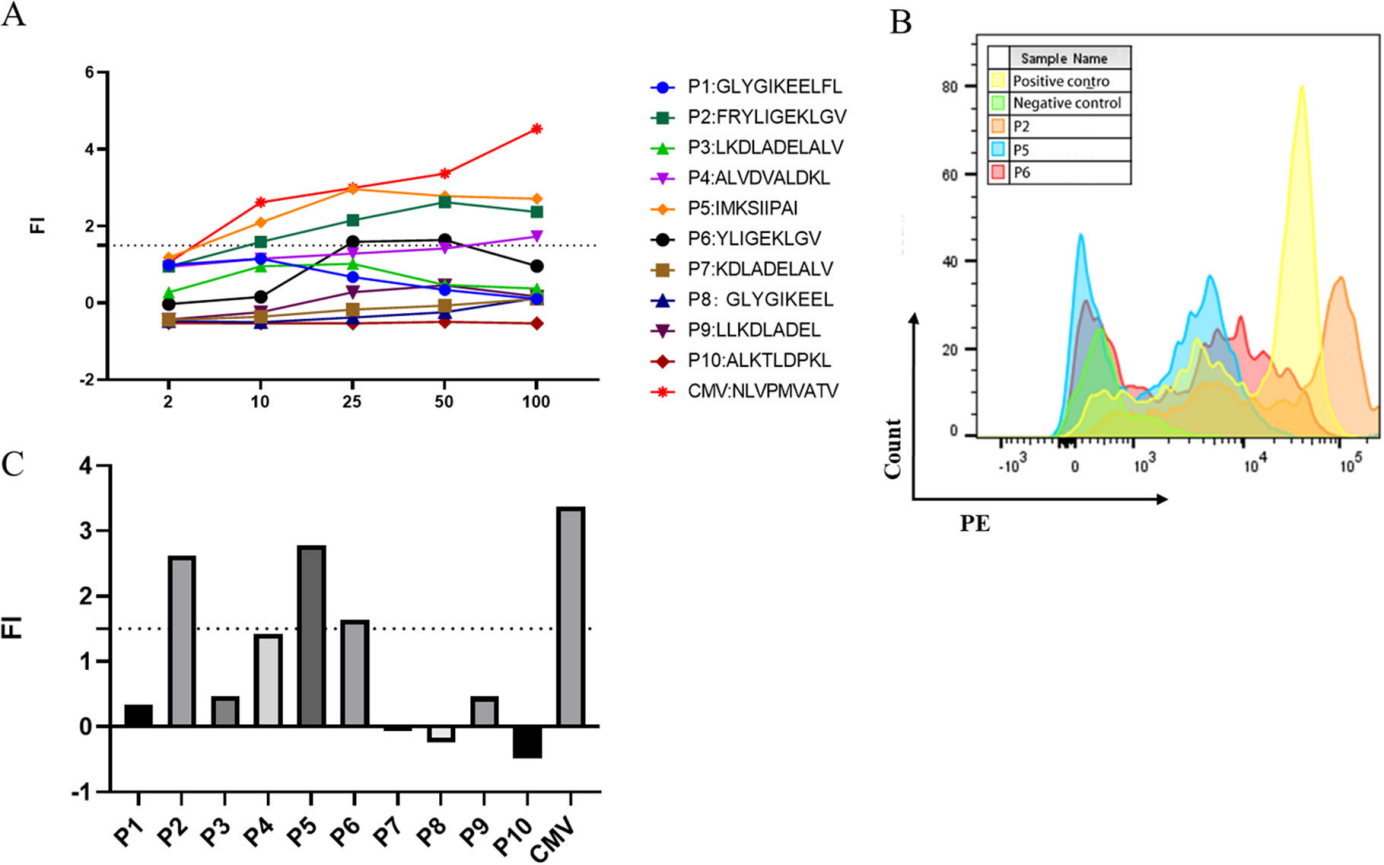
Screening of high-affinity peptides using the T2 affinity test and the concentration gradient test. **(A)** Fl of the 11 peptides at different concentrations. **(B)** Multiparameter flow cytometry scatter plot of P2, P5, and P6 for T2 cell affinity detection. **(C)** FI values of the 10 peptides at the concentration of 50 μg/mL.

Potential HLA-A2-restricted LDHC-derived peptides were predicted by the SYFPEITHI and NetMHC 4.0 servers. Ten peptides with the highest predicted scores were selected and synthesized for further study (**Table 1**). The purity of all peptides exceeded 95%. Subsequently, the T2 affinity assay was conducted to determine the affinity of all peptides. As shown in **Figure 2A**, peptides P2, P5, and P6 exhibited a fluorescence intensity (FI) >1.5, indicating a high affinity for the HLA-A2 molecule. **Figure 2B** showed the flow cytometry scatter plot of P2, P5, and P6 for T2 cell affinity detection, with positive and negative control. Finally, concentration gradient experiments were carried out on all peptides, and P2, P5, and P6 demonstrated the ability to stabilize HLA-A2 molecules at 50 μg/mL (**Figure 2C**). Therefore, P2, P5, and P6 at 50 μg/mL were selected for further analysis.

**Table 1:** Information on 10 candidate peptides.

No.	Pos	Peptides	Score_EL	%Rank_EL	Score_BA	%Rank_BA	Aff (nM)
P1	279–287	GLYGIKEEL	0.936597	0.029	0.816004	0.077	7.32
P2	170–180	FRYLIGEKLGV	0.797578	0.109	0.779587	0.145	10.86
P3	41–51	LKDLADELALV	0.885284	0.056	0.647099	0.604	45.53
P4	49–58	ALVDVALDKL	0.494336	0.399	0.500164	1.782	223.21
P5	116–124	IMKSIIPAI	0.477993	0.421	0.35347	4.244	1091.5
P6	172–180	YLIGEKLGV	0.885284	0.056	0.647099	0.604	45.53
P7	42–51	KDLADELALV	0.502242	0.39	0.629237	0.71	55.23
P8	279–287	GLYGIKEEL	0.498895	0.394	0.675307	0.482	33.55
P9	40–48	LLKDLADEL	0.459876	0.446	0.518937	1.581	182.18
P10	210–218	ALKTLDPKL	0.447889	0.462	0.580784	1.036	93.3

### 3.3 Docking of the HLA-A2 molecule and peptides

**Figure 3:**
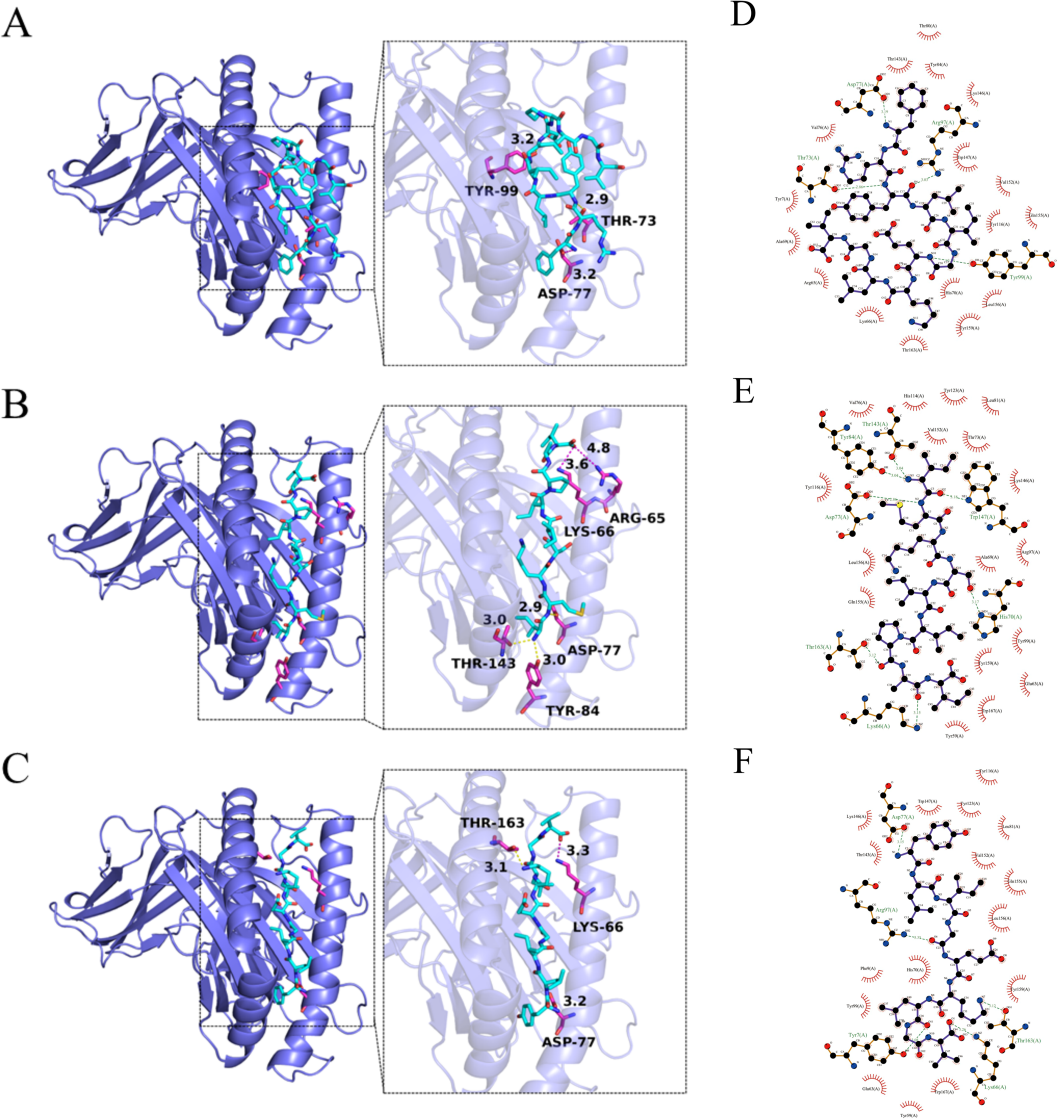
Interactions between the receptors **(A)** 3V5K -P2, **(B)** 3V5K -P5, and **(C)** 3V5K -P6. The 3V5K protein is represented as a slate cartoon model, the ligand is shown as a cyan stick, and their binding sites are shown as magenta stick structures. Non-polar hydrogen atoms have been omitted. The hydrogen bond, ionic interactions, and hydrophobic interactions are depicted as yellow, magenta, and green dashed lines, respectively. The 2D protein–peptide interaction diagrams **(D)** 3V5K -P2, **(E)** 3V5K -P5, and **(F)** 3V5K -P6. In the LigPlot diagrams, the green dashed lines represent hydrogen bonds. Red arcs indicate hydrophobic interactions, connecting the ligand to protein residues. Carbon atoms in the ligand are shown in black, oxygen atoms in red, and nitrogen atoms in blue. Protein residues are labeled with three-letter codes.

AutoDock Tools (ADT3) were used to prepare the receptor protein and ligand. The protein–ligand interactions were analyzed by AutoDock Vina (1.2.0). All functional residues were identified and classified according to their interactions. There are multiple groups of residues that are used to form interactions between the receptor protein and the ligand. With these interaction forces, the binding energy values of the 3V5K -P2, 3V5K -P5, and 3V5K -P6 complexes were −6.2 kcal/mol, −7.4 kcal/mol, and −8.1 kcal/mol, which indicated good performance (**Figures 3A–C**). To further analyze the protein–peptide interactions, we used the LigPlot software to generate 2D interaction plots, which illustrate the hydrogen bonds and hydrophobic protein–peptide interactions(**Figures 3D–F**).

### 3.4 CTLs induced by P6 can specifically lyse LUAD cells

**Figure 4:**
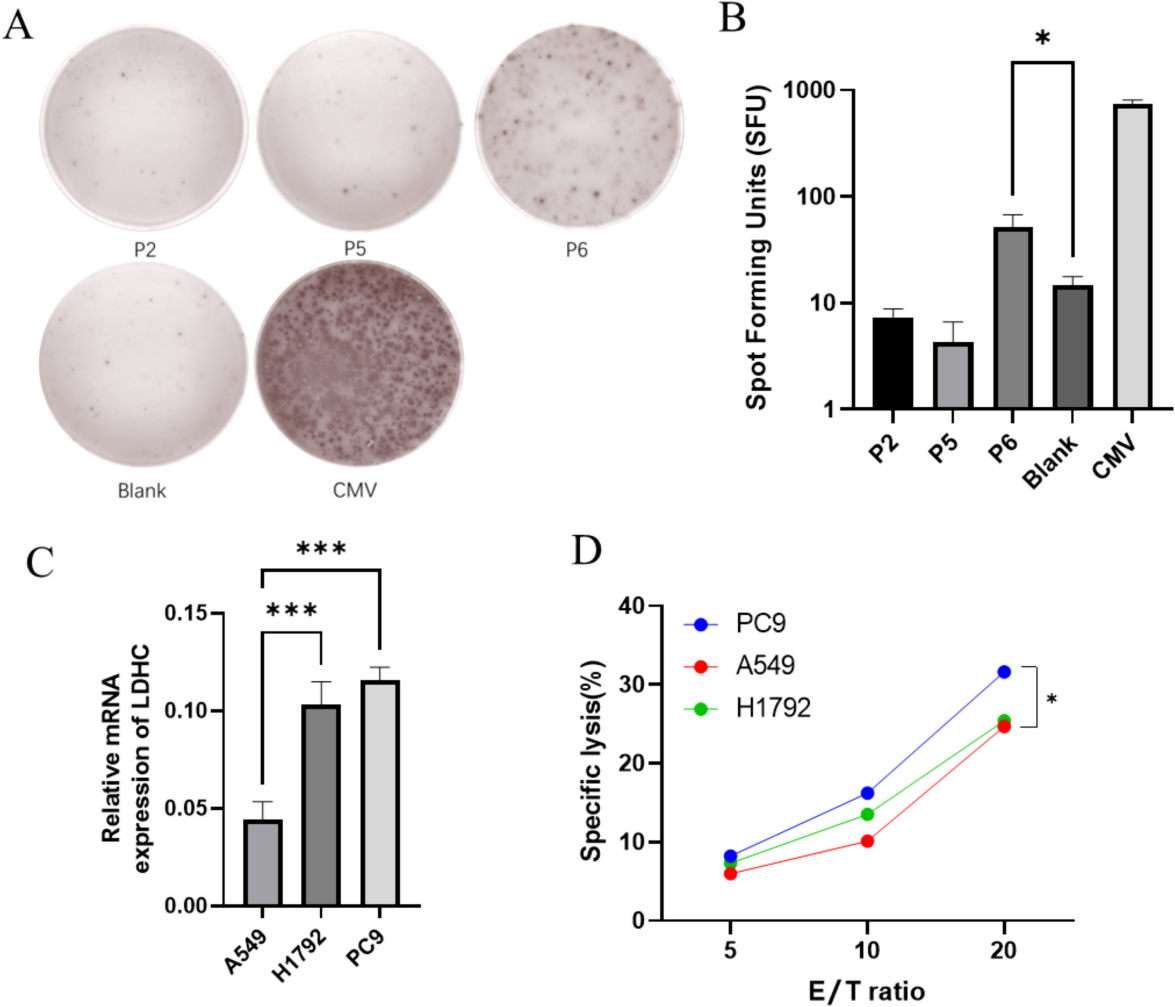
CTLs induced by P6 can specifically lyse LUAD cells. **(A, B)** P6 induced the highest number of IFN-γ secreting CTLs. **(C)** H1792 and PC9 had higher LDHC mRNA expression than A549. **(D)** CTLs induced by P6-loaded DCs had a higher specific lysis rate for PC9 than A549 (*p < 0.05; ***p < 0.001).

The ELISpot assay was utilized to evaluate the ability of high-affinity peptides identified in the T2 binding assays to induce CTLs to secrete IFN-γ. The number of IFN-γ secreting CTLs induced by different peptide-loaded DCs was quantified based on spot counts. Among the three candidate peptides, P6 induced the highest number of IFN-γ secreting CTLs compared with the blank control (*p* = 0.031) (**Figures 4A, B**). In order to select the target cell, the expression of LDHC in LUAD cells was detected by RT-qPCR. **Figure 4C** shows that H1792 and PC9 exhibited significantly higher LDHC mRNA expression levels than A549 (*p* = 0.0006 and *p* = 0.0002, respectively). Consequently, the LDH release assay showed that CTLs induced by P6-loaded DCs had a higher specific lysis rate for PC9 than A549 (*p* = 0.030) (**Figure 4D**).

## 4 Discussion

CTAs are tumor-associated antigens with high immunogenicity and restricted expression, which have been widely utilized to develop cancer vaccines (19, 20). However, the antitumor efficacy of CTA−based vaccines is limited to preclinical trials due to a lack of high immunogenicity, efficacy of antigen delivery and presentation processes, etc. (21, 22). LDHC is a kind of CTA that is reported to be abnormally expressed in LUAD and is a targetable CTA for immunotherapy (13). We aimed to investigate the efficacy of LDHC for immunotherapy in LUAD. In the present study, the LDHC recombinant protein was purified and co-cultured with the LUAD cell line PC9. Moreover, 10 high-affinity HLA-A2-restricted LDHC peptides were predicted, and three (P2, P5, and P6) were identified. In addition, the ELISpot assay and *in vitro* killing assay indicated that P6 can induce peptide-specific CTLs and demonstrated higher specific cytotoxicity against HLA-A2-positive and LDHC highly expressed cells.

There was promising evidence suggesting that LDHC may be a potential therapeutic strategy for cancer. It was reported that LDHC mRNA expression was upregulated in a wide range of human tumors, and LDHC protein was discovered in nearly all types of tumors examined (7). Data from previous studies indicated that the LDHC protein may participate in the humoral immune response, and the quaternary protein structure was crucial for the response of LDH-C4 antibodies (23, 24). Moreover, LDHC was found to promote tumor cell survival and invasion by maintaining the NAD^+^/NADH balance, which helps cancer cells resist oxidative stress and survive in harsh microenvironments (25). Accordingly, gene silencing of LDHC using RNA interference (RNAi) or CRISPR-Cas9 may inhibit cancer cell metabolism and proliferation (12, 26). Therefore, we investigated the tumorigenic effect of LDHC proteins on cancer cells by a co-culture model. In this study, we purified the LDHC recombinant protein covering the active site residues that are crucial for substrate recognition and catalytic reactions. Our analysis revealed that the LDHC recombinant protein promoted the migration and invasion of PC9 cells. As reported, peptides were derived from their respective protein antigens and then loaded onto MHC molecules through the antigen processing pathway (27). Accordingly, LDHC may be an ideal molecular target for peptide-based immunotherapy.

Peptide-based immunotherapy leverages peptides derived from LDHC to activate a patient’s immune system, particularly T cells. Active T cells recognize and attack cancer cells expressing LDHC and enhance the immune response against tumors, thereby achieving therapeutic effects against cancer (28, 29). In our study, we predicted and synthesized 10 HLA-A2-restricted LDHC peptides (P1–P10). Our study focused on the HLA-A2 type since it is the most frequent HLA molecule in the European/North American Caucasian population (27%) and the Arab population (25%–30%) (30, 31). In the present study, bioinformatic tools were used to predict LDHC-restricted peptides and perform molecular docking, which significantly improved experimental efficiency, optimized peptide design, and reduced experimental risks. Our results showed that specific CTLs induced by P6 (LDHC^172–180^, YLIGEKLGV) could secrete higher levels of IFN-γ and lyse HLA-A2/LDHC-positive LUAD cells. Similar to our findings, Thomas R. et al. have identified the immunogenic LDHC-derived peptides P11 (LDHC^41–55^) and P73 (LDHC^288–303^) that elicited CD8^+^ T-cell responses against HLA-A*0201/LDHC-positive breast cancer cells, demonstrating increased IFN-γ secretion and cytolytic activity (13). They also suggested that the majority of peptide-specific T cells exhibited an effector memory phenotype, which was crucial for sustained antitumor responses. Our study and other studies have highlighted the justification for LDHC as a targetable CTA in immunotherapy. By combining LDHC with neoantigens, it is expected that personalized vaccines can be developed to target specific tumor characteristics, thereby improving the precision and efficacy of treatment.

## 5 Conclusion

Nevertheless, our study has some limitations. First, the use of PBMCs from a single volunteer may limit the generalizability of the findings. Second, the study focuses on HLA-A*0201-restricted epitopes, which limits its applicability to patients with other HLA types. Third, the use of PBMCs from Chinese volunteers and cell lines of Caucasian origin may introduce ethnic-specific variability in immune responses. Future studies should include larger and more diverse patient cohorts to validate the findings and ensure broader applicability to different populations. Moreover, *in-vivo* studies and preclinical and clinical trials should be conducted to evaluate the therapeutic potential of the identified epitopes in a more physiologically relevant context.

This study showed that the LDHC recombinant protein could promote LUAD cell migration and invasion, and the HLA-A2-restricted LDHC peptide P6 (LDHC^172–180^, YLIGEKLGV) can efficiently induce CTLs to secrete IFN-γ and lyse LUAD cells. Based on our observations and evaluations, LDHC may serve as a targetable biomarker for peptide-based immunotherapy of LUAD.

## Data Availability

The raw data supporting the conclusions of this article will be made available by the authors without undue reservation.
