# Supplementary material for: Identification of new HLA-A*0201-restricted cytotoxic T lymphocyte epitopes from LDHC in lung adenocarcinoma
Source: Front Immunol. 2025 Apr 9;16:1564731. doi: 10.3389/fimmu.2025.1564731 (PMC12014551; doi:10.3389/fimmu.2025.1564731)
Supplement: Supplementary file 1 [file Table1.docx]

Supplementary Table 1 Structure Information of 5 models generated by GalaxyRefine server

| Model | GDT-HA | RMSD | MolProbity | Clash score | Poor rotamers | Rama favored |
| --- | --- | --- | --- | --- | --- | --- |
| Initial | 1 | 0 | 2.163 | 1.3 | 8 | 86.6 |
| MODEL 1 | 0.9826 | 0.302 | 2.103 | 17.7 | 0 | 94.8 |
| MODEL 2 | 0.9721 | 0.328 | 2.013 | 15.6 | 0 | 95.4 |
| MODEL 3 | 0.9773 | 0.332 | 2.019 | 15 | 0 | 95.1 |
| MODEL 4 | 0.9758 | 0.335 | 2.051 | 17.1 | 0 | 95.4 |
| MODEL 5 | 0.9773 | 0.323 | 2.053 | 15.6 | 0.7 | 94.8 |
